# Hepatitis E virus and renal injury: A review of pathogenesis, pathology, and clinical management

**DOI:** 10.1016/j.imj.2026.100259

**Published:** 2026-04-30

**Authors:** Jie Shen, Shujun Zhang

**Affiliations:** Key Laboratory of Infectious Diseases and Parasitic Diseases of Chongqing, Department of Infectious Diseases, The First Affiliated Hospital of Chongqing Medical University, Chongqing 400016, China

**Keywords:** Hepatitis E virus, Renal injury, Glomerulonephritis, Immune complex, Immunocompromised individuals

## Abstract

Hepatitis E virus (HEV), particularly *Orthohepevirus* A genotypes 1–4, is a major cause of acute viral hepatitis, with genotypes 3 and 4 also recognized as important zoonotic pathogens and increasingly associated with extrahepatic renal manifestations, which have emerged as a critical clinical challenge. Despite the growing body of case reports, a comprehensive synthesis of the biological mechanisms and standardized management for HEV-related kidney disease is still lacking. Biologically, HEV demonstrates distinct renal tropism, capable of completing its replication cycle within renal tubular epithelial cells and excreting infectious virions via urine. This review systematically explores the relationship between HEV and renal impairment, detailing the diverse renal pathologies ranging from acute kidney injury to immune-complex–mediated glomerulonephritis, such as IgA nephropathy, which manifests with greater frequency and severity in immunocompromised individuals. The underlying mechanisms are multifaceted, primarily involving the deposition of immune complexes containing the HEV open reading frame 2 capsid protein and host antibodies, which activate complement and drive glomerular injury, as well as potential direct cytopathic effects. Consequently, this article summarizes current clinical management, centering on antiviral therapy with ribavirin and the careful modulation of immunosuppressive regimens to provide a theoretical framework for clinicians.

## Highlights

• Zoonotic hepatitis E virus (HEV) demonstrates direct renal tropism, with evidence of productive replication in renal tubular epithelial cells and shedding of infectious virions in urine.
• HEV infection is linked to a spectrum of kidney injuries, particularly immune-complex-mediated glomerulonephritis such as IgA nephropathy and membranoproliferative glomerulonephritis.
• Immune-complex deposition is a key driver of injury, with HEV ORF2 capsid protein co-localizing with IgG and complement in glomeruli, independent of local viral replication.
• HEV triggers pro-inflammatory responses involving IFN-g and chemokine axes, amplifying renal damage through immune cell and renal epithelial crosstalk.
• Immunocompromised individuals are at highest risk for chronic HEV infection and severe, progressive HEV-related kidney disease.
• Management combines ribavirin antiviral therapy with immunosuppression reduction, though optimal strategies require further prospective studies.

## Graphical abstract

**Figure:**
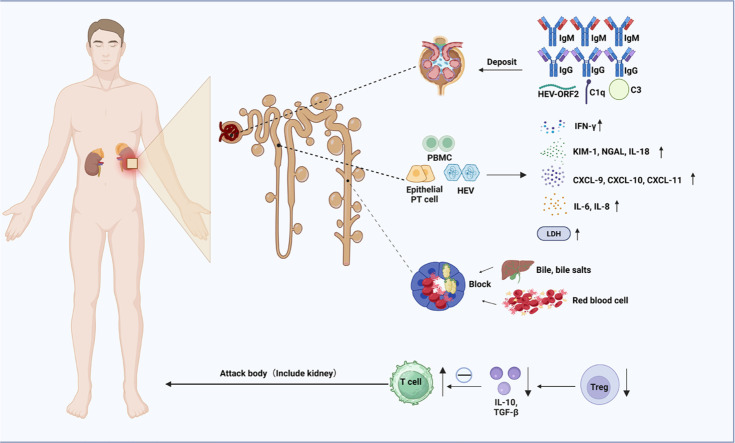

## 1 Introduction

Hepatitis E virus (HEV) is a positive-sense, single-stranded RNA virus in the family *Hepeviridae*, which contains the genera *Piscihepevirus* and *Orthohepevirus. Orthohepevirus* is divided into four species (A–D). *Orthohepevirus A* includes genotypes 1–8, among which genotypes 1–4 pose the greatest threat to human health.^1^^,^^2^ HEV-1 and HEV-2 infect only humans and are prevalent in developing regions. HEV-3 has a worldwide distribution, whereas HEV-4 is mainly found in China; both are zoonotic.^3^^,^^4^ Globally, HEV infection is now recognized as one of the leading causes of acute viral hepatitis.^5^^,^^6^ Hepatitis E (HE) was once regarded as an acute, self-limiting disease transmitted primarily via the fecal–oral route.^7^^,^^8^ More recent evidence shows that, in immunocompromised patients-including organ transplant recipients, HEV infection can progress to chronic disease. In addition, HEV is associated with high mortality in pregnant women and older adults, making it a serious public health concern.^9^, ^10^, ^11^ Beyond hepatic damage, HEV has been associated with a spectrum of extrahepatic manifestations involving the neurological, pancreatic, and hematologic systems, as well as renal involvement. Reported renal pathologies include membranoproliferative glomerulonephritis (MPGN), IgA nephropathy, membranous nephropathy (MN), and renal arteriosclerosis.^12^^,^^13^

The epidemiology of HEV-induced renal injury varies significantly based on the host’s immune status, the region underlying diseases as well as possible genetic factors. Globally, the anti-HEV IgG seroprevalence in the general population is estimated at 12.47%.^8^ In immunocompetent individuals, renal abnormalities reported during acute HEV infection are generally mild and self-limited, most frequently presenting as temporary proteinuria.^14^ Conversely, immune-mediated glomerular diseases subsequent to acute HEV infection seem to be uncommon and are not fully understood. Furthermore, in a Scottish cohort of 511 symptomatic patients with laboratory-confirmed HEV infection, 44 (8.6%) developed acute kidney injury (AKI),^15^ indicating that renal involvement may occur in a proportion of individuals with acute symptomatic HEV infection. In contrast, in immunocompromised individuals, particularly solid organ transplant (SOT) recipients, the risks of viral persistence and renal complications are markedly elevated. A systematic review revealed an HEV prevalence of approximately 20% in SOT recipients.^16^ In HEV-1 endemic regions such as Egypt, anti-HEV IgG seroprevalence was found to be 60.5% among patients with glomerulonephritis compared to 25% in healthy controls.^17^ Conversely, a prospective study from Germany, where HEV-3 predominates, did not demonstrate a statistically significant association between HEV seropositivity and overt glomerulonephritis, although anti-HEV–positive individuals exhibited numerically lower estimated glomerular filtration rate (eGFR) and higher serum creatinine levels.^18^

These conflicting observations highlight a fundamental gap in our understanding of HEV-associated renal injury and suggest that host immune status, virus genotype, geographic factors, and methodological discrepancies among studies may substantially influence the reported results. Moreover, the underlying mechanisms are probably distinct among renal morphologies rather than representing a single uniform process. Immune-mediated mechanisms may be more pertinent to glomerular diseases, whereas AKI may involve direct viral impacts and/or clinical complicating variables. Nonetheless, the relative contribution of these mechanisms remains incompletely understood. This review aims to systematically synthesize the available clinical and experimental evidence, critically evaluate sources of heterogeneity, and elucidate the pathogenic connections between HEV infection and various forms of renal injury, thereby providing a framework for future mechanistic studies and clinical management strategies.

## 2 HEV is capable of infecting and replicating in the urinary system

HEV is mainly spread through the fecal–oral route to infect the liver, but transmission can also occur via blood transfusion^19^, ^20^, ^21^ and transplanted organs in recipients, including the liver, lung, kidney, or heart.^22^, ^23^, ^24^ HEV RNA and HEV antigen (HEV-Ag) have also been detected in multiple extrahepatic tissues, such as the small intestine, brain, stomach, spleen, placenta, and kidneys,^25^, ^26^, ^27^, ^28^ indicating that HEV not only enters extrahepatic organs and cells but may also replicate therein. For example, Marion et al.^29^ demonstrated that HEV can infect intestinal epithelial cells and replicate within them before entering the liver in a quasi-enveloped state.

As shown in Table 1, five reported cases of chronic HEV infection following kidney transplantation from different countries were traced to donor-derived infection based on viral sequence concordance, infection timing, evaluation of other recipients from the same donor, and exclusion of alternative transmission routes, collectively supporting kidney transplantation as a route of HEV transmission.

**Table 1:** Possible HEV infection of recipients via transplanted kidney.

Cause of the donor’s death	Donor’s HEV viral load (log_10_ IU/mL)	Recipient’s basic medical history	Recipient’s liver function abnormalities	Time line	HEV genotype in the recipient	Recipient HEV IgM test	Recipient HEV IgG test	Recipient HEV RNA test and viral load	Treatment method	Whether antiviral therapy was given	Prognosis	Whether other recipients of donor grafts were infected	References
Acute subarachnoid hemorrhage	4.73	Focal segmental glomerulosclerosis, post-kidney transplantation	ALT↑	67 d	HEV-4	ND	ND	ND	Reduction of immunosuppressants, ribavirin therapy	Yes	Liver function and viral load tests tended to stabilize	Yes	^23^
Acute subarachnoid hemorrhage	4.73	chronic glomerulonephritis, post-kidney transplantation	ALT↑ ALP↑	54 d	HEV-4	ND	ND	ND	Reduction of immunosuppressants, ribavirin therapy	Yes	Liver function recovered, no detectable virus in the body	Yes	^23^
Cardiac arrest	4.9	Polycystic kidney disease, post-kidney transplantation	GGT↑	1 mo	HEV-3	Negative	Negative	Positive, 6.5 log_10_IU/mL	Ribavirin for 3 months and 4 months	Yes	Liver function returned to normal, no detectable virus in the body	None	^30^
Intracerebral hemorrhage	6.46	Hypertensive nephropathy	AST↑ ALT↑ GGT↑ ALP↑ Bilirubin↑	9 mo	HEV-3	Negative	Negative	Positive, 6.56 log_10_IU/mL	Reduction of immunosuppressants, ribavirin for 3 months	Yes	No detectable virus in the body	Yes	^31^
Intracerebral hemorrhage	6.46	Diabetes mellitus	AST ↑ ALT ↑ GGT ↑ ALP ↑ Bilirubin↑	11 mo	HEV-3	ND	ND	Positive, 6.78 log_10_IU/mL	Ribavirin therapy	Yes	Liver function recovered, no detectable virus in the body	Yes	^31^

*Notes:* Timeline denotes the interval from kidney transplantation to HEV diagnosis (d, days; mo, months). Donor HEV viral load is reported as log_10_ IU/mL. ↑ indicates a value above the upper limit of normal. IgM/IgG denote anti-HEV antibodies. “Immunosuppressant reduction” indicates dose reduction or temporary withdrawal. Prognosis reflects liver biochemistry and HEV RNA status at follow-up. “Other recipients infected” indicates whether recipients of other organs from the same donor were also infected. Bilirubin, total bilirubin.

*Abbreviations:* HEV, hepatitis E virus; ALT, alanine aminotransferase; AST, aspartate aminotransferase; ALP, alkaline phosphatase; GGT, Gamma-glutamyl transferase; ND, not determined.

Because HEV can be transmitted via transplanted kidneys, it is plausible—by analogy to intestinal epithelium—that HEV infects and replicates within renal epithelial cells, a notion supported by multiple experimental studies. *In vitro*, Nguyen et al.^32^ confirmed that the HEV-3 (Kernow strain) efficiently infects pig kidney cells, and Wang et al.^33^ detected HEV RNA in the kidneys of experimentally infected rabbits. HEV RNA and HEV-Ag were likewise found in the kidneys of Mongolian gerbils.^34^ Animal studies further demonstrated that both acute and chronic HEV infections can lead to viral RNA and antigens in urine, with a proportion remaining infectious.^35^^,^^36^

Clinically, Marion et al.^37^ analyzed urine from 24 transplant recipients with HEV infection and detected HEV RNA in 12 (50%) and HEV-Ag in 23 (96%). Complementary *in vitro* work by Wahid et al.^38^ using a variety of human renal cell lines (malignant and non-malignant) showed the renal cells are permissive to HEV entry, replication, and transmission, with replication levels in some renal cell lines comparable to-or exceeding-those in hepatocellular carcinoma cells. They confirmed that HEV can complete its full replication cycle in multiple renal cell lines, and that HEV subpopulations in vivo exhibit compartmentalized mutation characteristics.^38^

In renal transplant biopsies, Schmitz et al.^39^ localized HEV specifically to tubular epithelial cells using RNA-FISH, immunofluorescence, and electron microscopy. Moreover, the secretory open reading frame 2 (ORF2) capsid protein appears to be selectively taken up by renal cells from the bloodstream and excreted into urine.^40^ In rhesus macaques, Huang et al.^36^ noted glomerular changes characterized by widening of Bowman’s space and mild to moderate acute tubular necrosis despite normal renal function, with HEV-Ag detected in the cytoplasm of renal tubular epithelial cells.

Collectively, these findings indicate that HEV can infect the urinary system in animals and humans, complete a full replication cycle in renal cells—predominantly tubular epithelium—and shed infectious virions into urine, implicating the urinary tract as a potential transmission route.

## 3 HEV infection causes kidney damage

It is well established that the hepatitis B virus can infect the kidney and cause hepatitis B virus-associated nephropathy. By analogy, can HEV—which also shows renal tropism—damage the kidney as well Current evidence suggests that HEV infection can cause renal impairment of varying severity.^12^ In an Egyptian cohort, 5 of 31 patients (16%) with acute HEV-1 infection developed renal dysfunction, characterized by elevated blood urea nitrogen and creatinine; four patients experienced an eGFR decline to 60 mL/min/1.73 m².^41^ Among renal transplant recipients with HEV-3 infection, a retrospective study reported a decrease in glomerular filtration rate of approximately 5 mL/min during infection.^42^ HEV-4 infection has also been found to cause kidney damage in animal studies. Animal experiments further show that HEV-3 and HEV-4 replicate in rabbit renal tissue, with some HEV-4–infected animals developing elevated creatinine. Taken together, infections with HEV genotypes 1, 3, and 4 have been associated with kidney injury, and the risk and severity may vary by genotype and host context.

### 3.1 HEV-mediated renal damage in immunocompetent individuals

Among immunocompetent individuals, HEV infection is usually asymptomatic, and kidney involvement is rare. However, Zhang et al.^14^ found that 25% of immunocompetent patients with acute hepatitis E developed proteinuria, indicating that acute HEV infection is associated with renal injury even without immunosuppression. We summarize published reports of HEV-related kidney injury in immunocompetent populations in Table 2. HEV genotyping was seldom performed in these cases, largely because the disease in immunocompetent hosts is typically self-limiting and carries a favorable prognosis with conservative management.

**Table 2:** Case reports of renal injury caused by HEV in patients with normal immunity.

Patient information (Age & Sex)	Type of HEV-related renal injury	HEV genotype	Renal function at infection (creatinine, mg/dL)	Proteinuria	Treatment	Renal function after treatment (creatinine, mg/dL)	Antiviral therapy	Prognosis	Reference
50-year-old male	Cholestatic nephropathy	Not tested	5.6	No	Conservative treatment	1.2	No	Renal function improved	^43^
48-year-old male	MPGN	Not tested	3.63	No	Plasma exchange, corticosteroids	1.70–2.26	No	Renal function improved	^44^
25-year-old male	IgA nephropathy	Not tested	1.2	Yes	Corticosteroids	1.1	No	Renal function improved	^45^
56-year-old male	ARF (no biopsy)	Not tested	3.4	No	Hemodialysis	3.5	No	Cardiopulmonary arrest during treatment	^46^
3-year-old female	ARF (no biopsy)	Not tested	2.1	No	Conservative treatment	0.7	No	Renal function improved	^47^
38-year-old male	MN	Not tested	Not specified, normal	Yes	Corticosteroids	Not specified, normal	No	Normal renal function	^48^
34-year-old female	ARF (no biopsy)	Not tested	10.5	No	Corticosteroids	5.8	No	Renal function improved	^49^
76-year-old male	ARF (no biopsy)	Not tested	4	No	Ribavirin, 3 months	1–1.5	Yes	Renal function improved	^50^

*Abbreviations:* HEV, hepatitis E virus; MPGN, membranoproliferative glomerulonephritis; MN, membranous nephropathy; ARF, acute renal failure.

Across eight immunocompetent cases, acute renal failure without biopsy was the most common presentation (4/8), followed by biopsy-proven entities, including cholestatic nephropathy, MPGN, IgA nephropathy, and MN (1 case each). Baseline serum creatinine at presentation ranged from 1.2 to 10.5 mg/dL, and most patients experienced renal recovery with supportive care and/or corticosteroids; one patient required hemodialysis and died of cardiopulmonary arrest. Antiviral therapy with ribavirin was used in only one case. Notably, HEV genotyping was not performed in all reports, limiting genotype–phenotype inferences. Proteinuria was documented in a subset—particularly in glomerular diseases (IgA nephropathy and MN)—but was otherwise absent in most AKI presentations. Overall, the table suggests that HEV-associated kidney injury in immunocompetent hosts is heterogeneous, usually reversible, and infrequently treated with antivirals, with substantial gaps in virologic characterization.

### 3.2 HEV-mediated renal damage in immunocompromised individuals

Renal injury following HEV infection has been more frequently documented in immunocompromised individuals, including organ transplant recipients or those undergoing prolonged immunosuppression, and may also present with greater severity compared with immunocompetent individuals. Chronic HEV infection is more likely in this population,^51^^,^^52^ and viral persistence is associated with a higher risk of renal involvement. Clinically, HEV-related renal injury should be considered when immunocompromised patients present with elevated liver enzymes accompanied by unexplained hematuria, proteinuria, or a rise in serum creatinine.

Based on the 13 immunocompromised cases summarized in Table 3, HEV-related kidney injury most often presents as immune-complex–mediated glomerular disease—predominantly IgA nephropathy and membranoproliferative glomerulonephritis—with additional reports of MN and hyperbilirubinemia-associated AKI. Where genotyping was performed, genotype 3 predominated, with occasional genotype 1, and many cases lacked genotyping. Management typically combined ribavirin (commonly for 2–3 months but sometimes longer) with careful reduction or adjustment of immunosuppression; steroids or rituximab were used selectively for immune-mediated lesions, while conservative care was chosen in frail patients. Overall, roughly 8 of 13 patients improved or stabilized, whereas several progressed to end-stage kidney disease and a few died, often in the context of substantial comorbidities, underscoring the prognostic importance of early virologic control. These observations support routine HEV RNA testing in immunocompromised patients who develop liver enzyme abnormalities alongside new-onset hematuria, proteinuria, or rising creatinine, and they argue for timely antiviral therapy coupled with judicious immunosuppression modulation to mitigate renal injury.

**Table 3:** HEV infection in immunocompromised patients.

Patient information (Age & Sex)	Renal pathology or injury manifestation	HEV genotype	Renal function before infection (eGFR)	Renal function after infection (eGFR)	Treatment	Antiviral therapy	Renal function after treatment (eGFR)	Prognosis	Underlying disease	Reference
31-year-old male	Recurrent purpura/IgA nephropathy	Not tested	Not available	Not available	Ribavirin	Yes	Not available	Renal function improved	Post-kidney transplantation	^39^
33-year-old male	MPGN	HEV-3	56	41	Steroids, reduction of immunosuppressants	No	55	Renal function improved	Urinary tract malformation, post kidney transplantation	^42^
26-year-old male	Secondary IgA nephropathy	HEV-3	Not available	35	Ribavirin (3 months), no reduction of immunosuppressants	Yes	35	Renal function stable	IgA nephropathy, post kidney transplantation	^42^
40-year-old male	Secondary IgA nephropathy	HEV-3	53	39	Adjustment of immunosuppressants (dose and type)	No	35	Progressed to ESRD after 2 years	IgA nephropathy, post kidney transplantation	^42^
24-year-old male	MPGN	HEV-3	83	80	Rituximab (4 weeks)	No	37	Progressed to ESRD after 3 years	Alport syndrome, post-kidney transplantation	^42^
58-year-old male	Nonspecific renal injury	HEV-3	46.8	33.1	PEG-interferon (3 months)	No	27	Progressed to ESRD after 2 years	Primary sclerosing cholangitis, post liver transplantation	^42^
60-year-old male	MN	HEV-3	60	52	Ribavirin (3 months), adjustment of immunosuppressants	Yes	60	Renal function improved	Renal arteriosclerosis, Crohn’s disease, renal cell carcinoma, post-kidney transplantation	^53^
84-year-old male	Hyperbilirubinemia-associated acute kidney injury	Not available	Not available	62	Ribavirin (2 weeks)	Yes	Not available	Died 36 days after admission	Cutaneous lymphoma, hypertension	^54^
35-year-old male	Subacute kidney injury	Not available	Not available	< 20	Steroids; increased immunosuppression	No	Not available	Death due to enteritis	Post liver transplantation	^55^
46-year-old male	MPGN	HEV-3	80	41	Ribavirin (30 months), reduction of immunosuppressants	Yes	60	Renal function improved	Alport syndrome, third kidney transplantation	^56^
70+ year-old male	Subacute kidney injury	HEV-3	Not available	20–25	Conservative treatment	No	40–45	Renal function improved	Chronic kidney disease, alcoholic cirrhosis, cerebral infarction, hypertension, diabetes, hyperuricemia	^57^
78-year-old female	Immune-complex–mediated glomerulonephritis	HEV-1	Not available	Proteinuria 7.3 g/L	Ribavirin, reduction of immunosuppressants, and kidney transplantation	Yes	Proteinuria 2 g/L	Renal function improved	Diabetic nephropathy, post-kidney transplantation	^58^
28-year-old male	Mild renal impairment	Not tested	Not available	Cr: 1.86 mg/dL	Conservative treatment, no adjustment of immunosuppressants	No	Cr: 2.39 mg/dL	Renal function improved	Crescentic glomerulonephritis, post kidney transplantation	^59^

*Abbreviations:* eGFR, estimated glomerular filtration rate; ESRD, end-stage renal disease; PEG-interferon, pegylated interferon; Cr, creatinine.

### 3.3 Considering the variability in clinical studies of HEV-associated renal damage

The clinical evidence outlined above demonstrates substantial diversity in the manifestation, severity, and consequences of HEV-associated renal injury across studies and patient demographics. A primary factor contributing to this heterogeneity is probably the duration of infection. In immunocompetent individuals, HEV infection is typically acute and self-limited, which may account for the infrequency, mildness, and reversibility of renal problems. By contrast, in immunosuppressed patients—particularly solid organ transplant recipients—persistent HEV infection is more common and may promote prolonged viral replication, ongoing immune stimulation, and potentially viral evolution, thereby increasing the risk of sustained or exacerbated renal injury.

The host’s immunological status therefore seems to affect renal manifestations both directly and indirectly by influencing viral persistence. In immunocompetent individuals, the majority of documented occurrences transpired during acute HE and frequently improved with supportive treatment or brief corticosteroid therapy. Proteinuria was infrequently noted and mostly linked to biopsy-verified glomerular diseases such as IgA nephropathy and MN, despite numerous cases of AKI lacking histological validation. In contrast, immunocompromised patients, especially solid organ transplant recipients, have been more frequently documented to experience renal complications, including immune-complex–mediated glomerular disease and progression to chronic kidney disease or end-stage renal disease.

Additional factors may also contribute to the reported variability. The viral genotype may affect tissue tropism, replication kinetics, and immunogenicity, although the relationships between genotype and phenotype are insufficiently defined. Methodological inconsistencies across studies, including limited use of renal biopsy, reliance on serological markers rather than HEV RNA testing, and heterogeneous definitions of renal outcomes, further complicate interpretation. Clinical confounders such as liver dysfunction, hyperbilirubinemia, nephrotoxic drugs, hemodynamic instability, and pre-existing kidney disease may also contribute to renal dysfunction, particularly in immunocompromised patients.

In summary, HEV-associated renal injury should not be regarded as a single entity, but rather as a heterogeneous spectrum shaped primarily by infection chronicity and host immune status, with additional contributions from viral factors, study methodology, and clinical context. Future studies should apply standardized diagnostic criteria, incorporate systematic HEV RNA testing and genotyping, and stratify analyses according to immune status and infection duration to better define the true renal pathogenicity of HEV.

## 4 Mechanisms of HEV-associated renal damage

### 4.1 Immune-complex deposition pathway

At the molecular level, HEV-associated immune-complex–mediated renal injury is unlikely to be a passive or nonspecific process. Instead, accumulating evidence suggests that it reflects selective biochemical interactions between circulating HEV antigens—particularly the secreted form of the secreted ORF2 (sORF2)—and the unique physicochemical properties of the glomerular basement membrane (GBM). sORF2 is abundantly released into the circulation during active infection, is highly immunogenic, and readily forms antigen–antibody complexes with anti-HEV IgG. The GBM is enriched in negatively charged heparan sulfate (HS) proteoglycans, creating an anionic microenvironment that may preferentially trap cationic regions of ORF2-containing immune complexes. This selective retention provides a mechanistic basis for the observed glomerular deposition of HEV antigens, subsequent complement activation, and immune-complex–mediated glomerular injury.

**Fig. 1:**
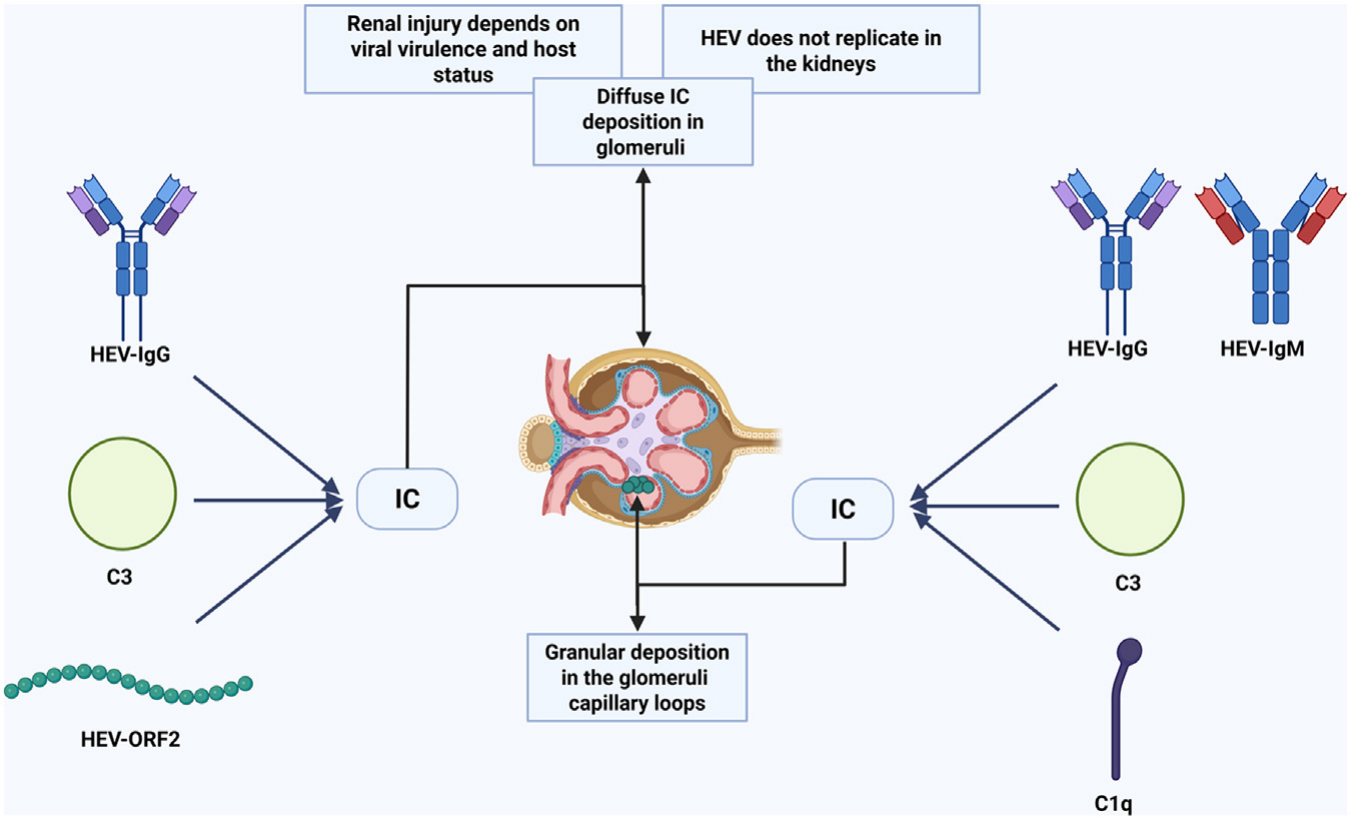
Immune-complex deposition pathway. Rather than direct renal replication, circulating HEV ORF2 antigen can form immune complexes with anti-HEV IgG and C3, leading to diffuse IC deposition in the glomeruli. Alternatively, C1q may form immune complexes with anti-HEV IgG/IgM and C3, resulting in granular deposition in the glomerular capillary loops. HEV infection may lead to MPGN and cryoglobulinemia via immune complex formation. Renal injury severity depends on viral virulence and host status. Created in BioRender. shen, J. (2026) https://BioRender.com/zwczyig. *Abbreviations*: HEV, hepatitis E virus; ORF2, open reading frame 2; IC, immune-complex; MPGN, membranoproliferative glomerulonephritis.

Consistent with this model, clinical and histopathological evidence strongly links HEV infection to immune-complex formation and deposition in the kidney (Fig. 1). One clinically relevant correlate of this process is cryoglobulinemia, a pathological condition in which circulating immunoglobulins and/or immune complexes precipitate at low temperatures and re-dissolve upon rewarming.^60^ Horvatits et al.^61^ reported cryoglobulins in 7% of patients with acute HEV infection and in 27% of those with chronic infection. Patients with cryoglobulinemia had significantly higher serum creatinine levels (1.6 mg/dL [interquartile range: 1.3–2.8] vs. 0.9 mg/dL [interquartile range: 0.8–1.2], *p* = 0.007). Guinault et al.^44^ provided the first evidence that HEV directly contributes to cryoglobulin formation and immune-complex–mediated glomerular injury: during active infection (positive anti-HEV IgG/IgM and HEV RNA), the patient had type II cryoglobulinemia (reported as monoclonal IgGκ in this case), and renal biopsy demonstrated MPGN with granular IgG, IgM, and complement (C3, C1q) deposits along glomerular capillary loops. Molecular analysis of the cryoprecipitate identified HEV RNA together with anti-HEV IgG/IgM, providing the first confirmation that HEV antigen–antibody complexes constitute the core of cryoglobulins. Following plasma exchange combined with glucocorticoid therapy, HEV RNA became undetectable in both serum and cryoprecipitate, cryoglobulinemia resolved, and renal function improved markedly; on follow-up biopsy, glomerular inflammation was clearly reduced. However, the study did not assess whether HEV-related cryoprecipitate deposits were present in glomerular capillaries.^62^^,^^63^

Complementing the above findings, Leblond et al.^64^ presented a case series of four patients demonstrating direct glomerular deposition of HEV ORF2 protein and immune-complex formation in three patients with acute HE on cirrhosis and one kidney transplant recipient with chronic HEV infection. Notably, in the transplant recipient, MPGN developed despite the absence of detectable intrarenal HEV RNA replication, strongly implicating circulating antigens rather than local viral replication as the primary driver of renal injury. Quantitative imaging revealed high levels of IgG–ORF2 co-localization at both the whole-glomerulus and high-resolution field levels, with robust Pearson’s correlation coefficients of 0.838 ± 0.039 (*n* = 25 glomeruli; *p* < 10^−10^) for whole-glomerulus imaging and 0.668 ± 0.138 (*n* = 16; *p* < 10^−10^) for high-resolution fields.^64^ Additionally, Manders coefficients for 20× images were M1 = 0.84 ± 0.08 and M2 = 0.83 ± 0.09 (*n* = 25 glomeruli). Complement (C3) was also observed within glomerular lesions, further supporting the role of immune-complex formation in renal injury.^40^^,^^64^, ^65^, ^66^, ^67^ Semiquantitative ORF2 immunohistochemistry revealed a more pronounced accumulation of glomerular ORF2 in chronic infections (intensity score +++) compared to acute cases (intensity score +), hence endorsing a load-dependent immune-complex/complement-mediated damage pathway.

The selective deposition of circulating antigens within the glomerulus likely has a clear molecular basis. The GBM contains a dense polyanionic matrix in which negatively charged HS chains provide a physical scaffold for antigen retention through electrostatic interactions with cationic domains of the ORF2 protein.^68^ The initial attachment of ORF2 capsid protein appears to be mediated by binding to cell-surface heparan sulfate proteoglycans, a process highly dependent on specific sulfation patterns—particularly 6-O sulfation—within the glycosaminoglycan chains.^69^ Under pathological conditions, this interaction landscape may be further remodeled. Ferreras et al.^70^ demonstrated that chronic kidney injury is associated with downregulation of the HS-modifying enzyme HS3ST1, resulting in reduced 3-O sulfation. Although the direct impact of altered 3-O sulfation on HEV binding remains to be elucidated, the alteration of 6-O sulfation patterns in this context remains to be elucidated. We hypothesize that this “vacancy”, together with preserved or pathologically increased anionic motifs (e.g., 2-O sulfation), creates a microenvironment that favors ORF2 accumulation. Such retention would enhance local immune-complex formation and amplify complement activation within glomeruli.

Beyond passive trapping of circulating HEV antigens within the glomerulus, a misdirected immune response driven by molecular mimicry may represent an additional, though still unproven, mechanism of renal injury.^71^ In this context, antiviral B- or T-cell responses elicited by HEV antigens may cross-react with structurally similar host epitopes present in glomerular or vascular compartments, thereby converting a virus-directed immune response into tissue-damaging autoimmunity.^72^^,^^73^ This process elucidates why renal injury may persist or progress in the absence of detectable intrarenal viral reproduction, and why the extent of tissue damage may surpass expectations based only on antigen deposition. Molecular mimicry has been suggested as an indirect immunopathological explanation for HEV-related extrahepatic symptoms, while direct proof in renal illness is still absent. We regard mimicry not as a substitute for the ORF2 immune-complex concept, but as a possible enhancer of complement activation, inflammatory cell recruitment, and prolonged glomerular injury following the deposition of immune complexes.^64^

Collectively, these data support a unified model in which secreted, non-infectious ORF2 acts as a circulating immunogenic antigen that binds host IgG, preferentially deposits within the glomerular basement membrane via charge- and sulfation-dependent interactions, activates the classical complement pathway, and precipitates immune-complex–mediated glomerulopathy. Future studies should directly assess cryoglobulin and ORF2 co-localization within glomerular capillaries and clarify the causal contribution of complement activation to renal injury progression.

### 4.2 HEV-mediated immune activation and inflammation

Beyond immune-complex deposition, HEV-associated renal injury can also arise from innate immune activation and inflammatory signaling triggered by viral sensing, even in the absence of detectable immune-complex deposition. In renal tubular epithelial cells and infiltrating immune cells, HEV RNA is likely recognized by pattern recognition receptors (PRRs), including cytosolic RIG-I-like receptors and endosomal Toll-like receptors such as TLR3 and TLR7. Engagement of these sensors initiates downstream signaling cascades—most prominently NF-κB and JAK/STAT pathways—leading to the induction of interferons, chemokines, and pro-inflammatory mediators. This signaling framework provides a mechanistic link between HEV infection and the renal inflammatory phenotypes observed in both experimental models and clinical cases.

**Fig. 2:**
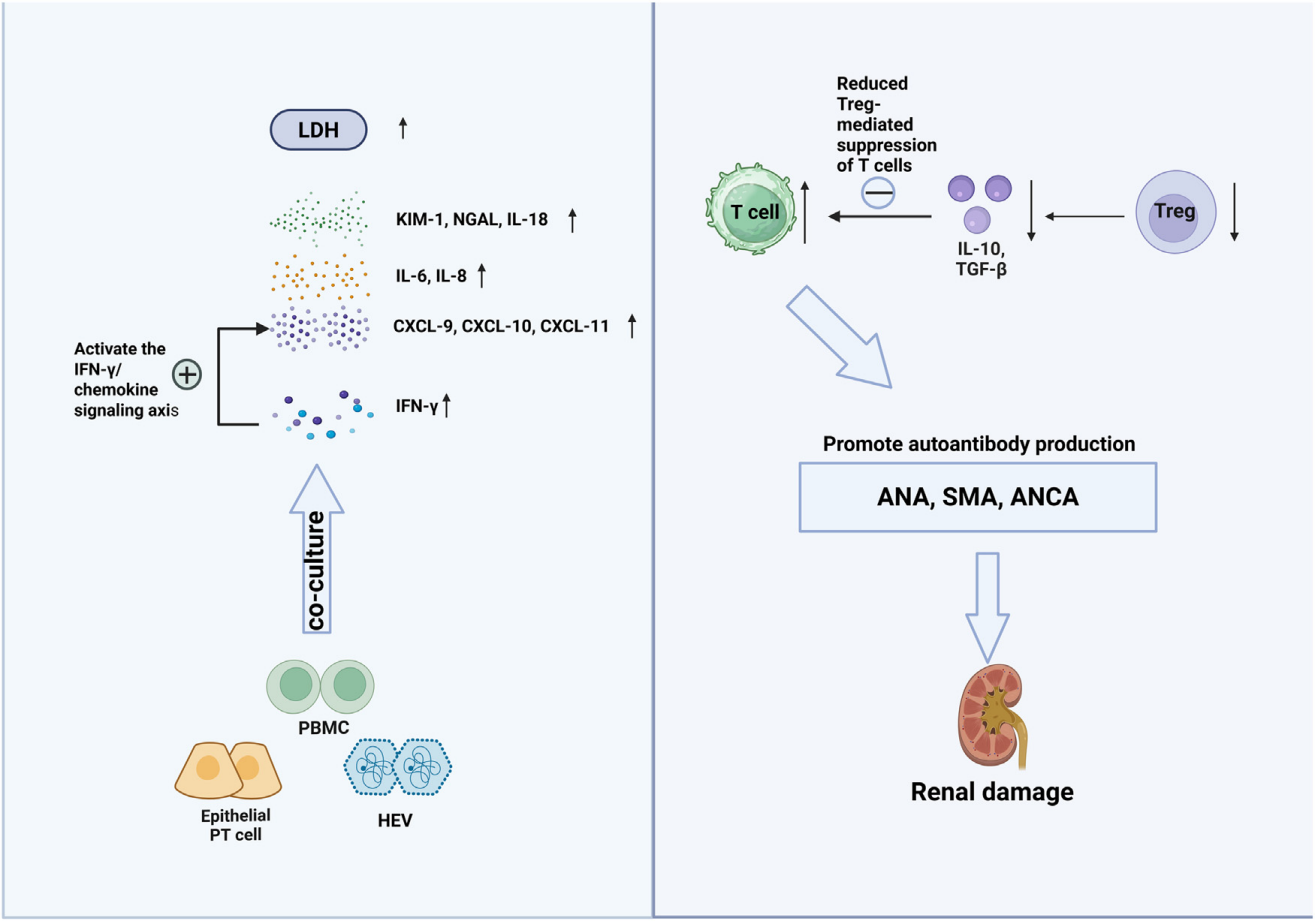
HEV-mediated immune activation and inflammation. HEV infection of proximal tubular epithelial cells, when co-cultured with PBMCs, induces chemokines (CXCL-9/10/11) via the IFN-γ signaling axis and produces more proinflammatory cytokines (e.g., IL-6, IL-8, IL-18), leading to epithelial injury. In some patients, reduced Treg function diminishes suppression of effector T cells, resulting in autoantibody production (ANA, SMA, ANCA) and subsequent renal injury. Created in BioRender. shen, J. (2026) https://BioRender.com/5cbjg5u. *Abbreviations*: LDH, lactate dehydrogenase; KIM-1, kidney injury molecule-1; NGAL, neutrophil gelatinase-associated lipocalin; IL, interleukin; CXCL, C-X-C motif chemokine ligand; IFN-γ, interferon-gamma; PBMC, peripheral blood mononuclear cell; PT cell, proximal tubule cell; Treg, regulatory T cell; TGF-β, transforming growth factor-beta.

Supporting the existence of immune-complex–independent mechanisms, Verschuuren et al.^49^ reported a case of acute renal failure following HEV infection in which renal biopsy revealed no vascular abnormalities, and immunofluorescence was negative for immunoglobulins and complement components, indicating that alternative inflammatory pathways may drive renal injury in certain settings (Fig. 2).

Experimental studies further delineate how immune–epithelial crosstalk amplifies HEV-induced renal inflammation. HEV activates both innate and adaptive immunity, triggering systemic inflammatory responses that exacerbate renal injury.^74^^,^^75^ In a Mongolian gerbil model, intraperitoneal HEV infection resulted in marked renal infiltration by lymphocytes and macrophages, whereas control animals showed no significant histopathological changes.^76^ *In vitro*, HEV-infected CD10⁺/CD13⁺ human proximal tubule epithelial cells exhibited minimal inflammatory responses when cultured alone. However, co-culture with autologous peripheral blood mononuclear cells significantly amplified inflammation signals, with increased production of pro-inflammatory cytokines (IL-6, IL-8), chemokines (CXCL9, CXCL10, CXCL11), and renal injury markers (KIM-1, NGAL, IL-18), accompanied by elevated lactate dehydrogenase release as a marker of cell damage.^75^^,^^77^ Notably, IFN-γ emerged as a central mediator in this immune–epithelial interaction. During peripheral blood mononuclear cell co-culture, IFN-γ levels increased significantly (*p* ≤ 0.001), consistent with activation of an IFN-γ–chemokine axis that promotes leukocyte recruitment, sustains local inflammation, and exacerbates tubular injury. Within the signaling framework proposed above, IFN-γ likely acts downstream of PRR activation through JAK/STAT signaling, reinforcing chemokine expression and inflammatory amplification loops in the renal microenvironment.

At the same time, HEV infection can engage counter-regulatory immune mechanisms. Several studies^78^, ^79^, ^80^ have documented expansion of regulatory T cells (Tregs) during acute HEV infection, which may limit excessive immunopathology through IL-10 and TGF-β production and contribute to the self-limiting nature of infection in immunocompetent hosts. Clinically, rapid remission of primary MN following HEV infection has been reported, potentially reflecting HEV-induced Treg expansion.^79^^,^^81^ Conversely, a prospective study found that nearly half of acute HEV cases developed autoantibodies (e.g., ANA, SMA, ANCA), indicating that HEV can also trigger an autoimmunity-like activation state that may contribute to organ injury.^82^ In immunocompromised patients, impaired Treg-mediated restraint and altered immune balance may favor unchecked inflammatory signaling, thereby increasing susceptibility to renal and extrahepatic injury.^80^^,^^83^

Taken together, these findings support a model in which HEV-associated renal injury arises not only from immune-complex deposition but also from PRR-driven immune activation, characterized by NF-κB and JAK/STAT signaling, IFN-γ–dependent chemokine cascades, and dynamic immune regulation. The balance between inflammatory amplification and regulatory control appears to be a critical determinant of renal outcome across different host immune states.

### 4.3 HEV aggravates kidney injury via systemic complications

In addition to direct renal involvement and immune-mediated injury, HEV infection can indirectly aggravate kidney damage through systemic complications arising from severe hepatic dysfunction, cholestasis, hematologic abnormalities, and immune dysregulation. In these settings, renal injury is not driven by local viral replication or immune-complex deposition within the kidney, but rather represents a secondary consequence of profound systemic disturbances triggered by HEV infection. This indirect pathway is particularly relevant in patients with acute liver failure, advanced cirrhosis, or severe extrahepatic manifestations, where renal vulnerability is markedly increased.

Severe liver injury caused by HEV infection, particularly acute liver failure, can lead to hepatorenal syndrome (HRS).^84^ Superinfection with HEV in patients with underlying chronic liver disease further amplifies this risk. For example, patients with chronic hepatitis B who acquire HEV infection have been shown to exhibit a significantly higher incidence of HRS compared with those with hepatitis B alone.^85^ The pathophysiology of HRS involves marked splanchnic vasodilation, reduced effective arterial blood volume, activation of vasoconstrictive systems, and subsequent renal hypoperfusion, ultimately leading to functional renal failure despite structurally intact kidneys.^86^

In the context of severe cholestasis, bile cast nephropathy represents another important mechanism of HEV-associated kidney injury. Extreme hyperbilirubinemia can exert direct tubular toxicity and promote intraluminal bile cast formation, resulting in acute tubular injury. Nayak et al.^43^ described a patient with acute HE whose total bilirubin level rose to 33.9 mg/dL, followed by non-oliguric AKI. Renal biopsy revealed diffuse acute tubular injury with extensive bile casts, confirming bile cast nephropathy as the underlying mechanism.

HEV infection has also been implicated in hematologic and immune-mediated systemic disorders that secondarily impair renal function. Monoclonal gammopathy of renal significance has been reported following acute HEV-related liver injury, with renal biopsy demonstrating light-chain–restricted casts, interstitial inflammation, and fibrosis, and serum studies confirming a monoclonal component.^87^ In addition, HEV can trigger glucose-6-phosphate dehydrogenase deficiency–related hemolysis and thrombotic thrombocytopenic purpura, leading to severe intravascular hemolysis, marked hyperbilirubinemia, microvascular injury, and acute kidney failure that may progress to irreversible renal damage.^46^^,^^88^, ^89^, ^90^, ^91^

**Fig. 3:**
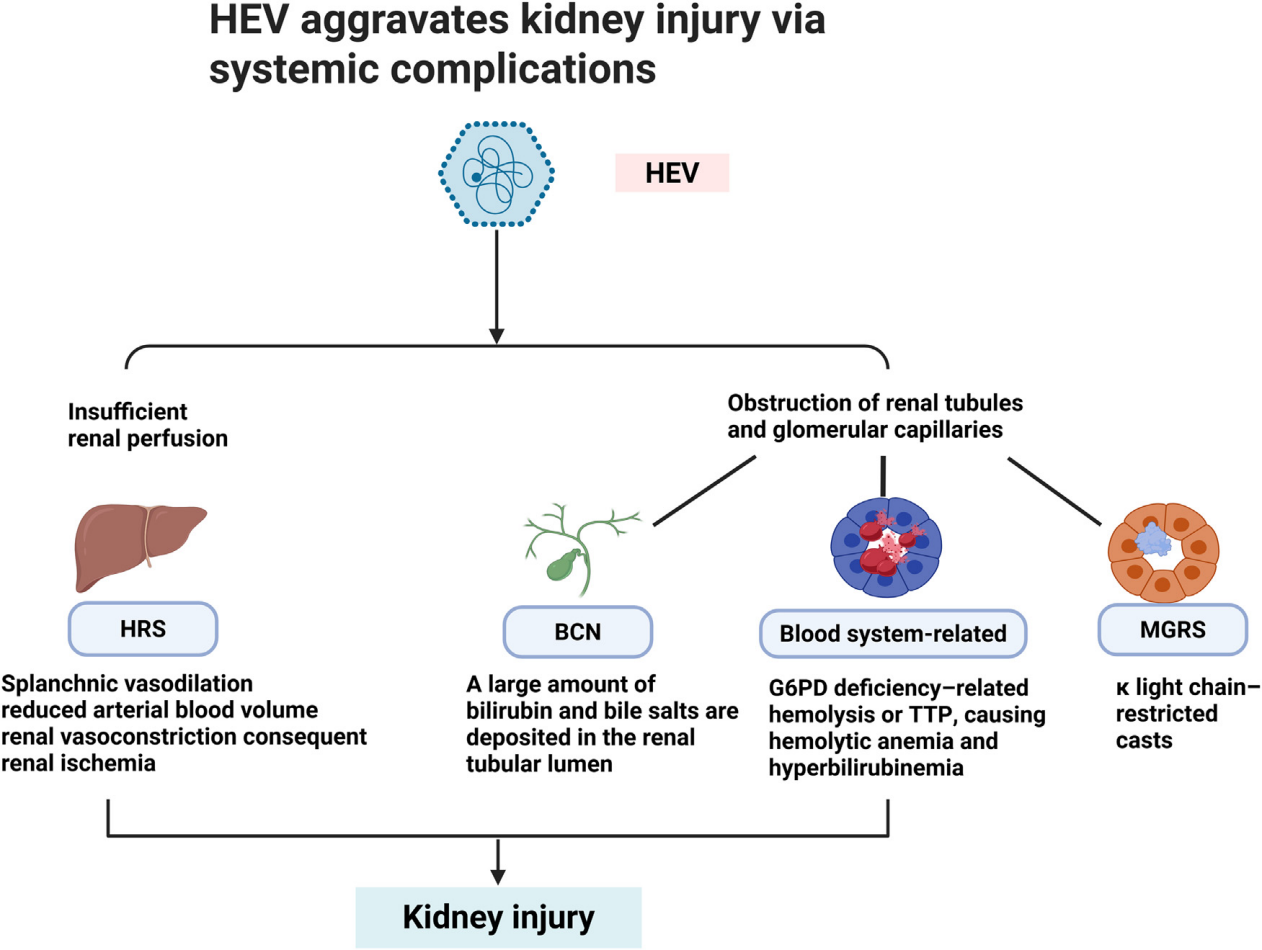
HEV aggravates kidney injury via systemic complications. HEV can aggravate renal injury through systemic pathways, including HRS, BCN, hematologic complications such as G6PD deficiency–related hemolysis or TTP, and MGRS. Created in BioRender. shen, J. (2026) https://BioRender.com/61l7u2i. *Abbreviations:* HEV, hepatitis E virus; HRS, hepatorenal syndrome; BCN, bile cast nephropathy; G6PD, glucose-6-phosphate dehydrogenase; TTP, thrombotic thrombocytopenic purpura; MGRS, monoclonal gammopathy of renal significance.

Collectively, these observations underscore that HEV-associated renal injury extends beyond direct viral or immune-mediated mechanisms and includes a spectrum of systemic, secondary pathways. Recognition of these indirect mechanisms is clinically critical, as renal injury in this context may be reversible with timely management of hepatic failure, cholestasis, or hematologic complications, in addition to antiviral therapy. Accordingly, clinicians should maintain a high index of suspicion for HEV infection in patients presenting with acute hepatic decompensation, unexplained hemolysis or thrombotic microangiopathy, and concomitant renal dysfunction, and should implement early supportive and etiologic interventions to mitigate kidney injury (Fig. 3).

## 5 Treatment

The management of HEV infection varies according to the patient’s immune status, etiology, and disease course. Regarding the kidney damage caused by different factors in HE: Immune-complex–mediated damage is treated mainly with antiviral therapy; and secondary renal injury due to severe hematologic or immune disorders, hepatic damage, or cholestasis requires primarily supportive measures, including alleviation of cholestasis, maintenance of hemodynamic stability, and avoidance of nephrotoxic agents. In immunocompetent patients with acute HEV infection, symptomatic and supportive therapy is usually sufficient, and most recover spontaneously. In immunocompromised patients, such as SOT recipients or those with chronic kidney disease, initial management typically includes a cautious reduction of immunosuppression, when feasible. If immunosuppression cannot be safely reduced or if chronic HEV (persistently detectable HEV RNA ≥ 3 months) is present, antiviral therapy is indicated, with ribavirin as the preferred first-line agent.^92^ It is crucial to adjust the dose based on the patient’s kidney function to avoid drug accumulation, which may lead to adverse reactions like hemolytic anemia. Based on the population pharmacokinetic and pharmacodynamic analysis of chronic HEV infection in SOT recipients, the recommended dosing regimen for ribavirin is as follows: For patients with eGFR ≥ 60 mL/min/1.73 m², the recommended dose is 600 mg per day for 180 days. For those with eGFR between 30 and 59 mL/min/1.73 m², the recommended dose is 400 mg per day for 180 days. For patients with eGFR ≤ 30 mL/min/1.73 m², the recommended dose is 200 mg per day for 180 days.^93^ This regimen achieves good efficacy while minimizing toxicity, particularly reducing the risk of severe anemia. Many patients achieve viral clearance and renal stabilization/improvement with this strategy. Ribavirin monotherapy for three months is commonly used, and treatment is considered successful if a sustained virological response is achieved (HEV RNA is undetectable in both plasma and stool six months after therapy).^94^ However, the optimal duration of ribavirin treatment remains unclear. As for pegylated interferon-α (Peg-IFN-α), which establishes an antiviral state by activating the JAK-STAT signaling pathway through the combination of cell surface receptors, thereby inducing the expression of multiple interferon-stimulated genes.^95^^,^^96^ But its strong immune activation significantly increases the risk of organ rejection in transplant recipients. Literature reports indicate that about 15% of patients undergoing interferon treatment experience acute transplant rejection. Therefore, Peg-IFN-α should generally be avoided in kidney transplant recipients and other non-liver organ transplant recipients. Even in liver transplant patients, Peg-IFN-α should only be used as a last resort after ribavirin failure, and its use should be extremely cautious, with close monitoring of organ function.^92^^,^^97^

## 6 Outlook

Moving forward, research must transition from descriptive clinical observations to hypothesis-driven investigations to address the complex HEV-renal problem. A primary challenge lies in direct viral cytopathic effects from indirect immune-mediated injury across diverse populations. Specifically, it remains to be determined whether the high intrarenal viral load observed in immunosuppressed patients is the primary driver of tubular damage, or if the injury is predominantly governed by the systemic inflammatory crosstalk between immune cells and the renal epithelium via the IFN-γ/chemokine axis. Furthermore, the discovery of glomerular sORF2 deposition in the absence of local replication raises a critical question: Is this antigen an inert bystander or a dynamic driver of persistent inflammation Investigating whether sORF2 modulates podocyte function or activates the complement cascade will be pivotal. To validate these molecular pathways, the field should move toward advanced experimental platforms, such as human kidney organoids and kidney-on-a-chip technology, which can more accurately simulate the interaction between HEV and the renal basement membrane matrix, including the 6-O/3-O sulfation patterns of heparan sulfate proteoglycans. Finally, prospective cohorts integrating protocolized biopsies and multi-omics are essential to clarify which patients benefit most from early antiviral intervention versus immunosuppression modulation, ensuring that virological clearance translates effectively into long-term renal preservation.

## CRediT authorship contribution statement

**Jie Shen:** Writing – original draft, Visualization, Software, Conceptualization. **Shujun Zhang:** Writing – review & editing, Writing – original draft, Supervision, Funding acquisition, Conceptualization.

## Informed consent

Not applicable.

## Organ donation

Not applicable.

## Ethical statement

Not applicable.

## Data availability

Data sharing is not applicable to this article as no datasets were generated or analysed.

## Animal treatment

Not applicable.

## Generative AI

We confirm that AI-assisted technologies were only used for language polishing, and all scientific content, data, and interpretations were created and verified by the authors.

## Funding

This work was supported by Senior Medical Talents Program of Chongqing for Young and Middle‐aged, grant number 2019‐181 and Joint Project of Chongqing Health Commission and Science and Technology Bureau, grant numbers: 2019MSXM076 and 2020FYYX007.

## Declaration of competing interest

The authors declare that they have no known competing financial interests or personal relationships that could have appeared to influence the work reported in this paper.
